# Differences by Physician Seniority in Race and Ethnicity and Insurance Coverage of Treated Patients

**DOI:** 10.1001/jamanetworkopen.2023.47367

**Published:** 2023-12-13

**Authors:** Hannah T. Neprash, David C. Chan, Ateev Mehrotra, Michael L. Barnett

**Affiliations:** 1 Division of Health Policy and Management, University of Minnesota School of Public Health, Minneapolis; 2 Department of Health Policy, Stanford University, Stanford, California; 3 Department of Veterans Affairs, Palo Alto, California; 4 Department of Health Care Policy, Harvard Medical School, Boston, Massachusetts; 5 Department of Health Care Policy and Management, Harvard T. H. Chan School of Public Health, Boston, Massachusetts; 6 Division of General Internal Medicine and Primary Care, Brigham and Women’s Hospital, Boston, Massachusetts

## Abstract

This cross-sectional study investigates the share of patients who were members of racial and ethnic minority groups or Medicaid enrollees by physician seniority.

## Introduction

In a discussed but little-studied practice, senior physicians preferentially treat patients with more generous commercial insurance, whereas junior physicians treat more patients with Medicaid.^1,2^ Medicaid has less generous payments but disproportionately includes Black or Hispanic patients. A 2-tiered system by physician seniority could act as an institutional mechanism for racial and economic segregation, limiting access to more experienced physicians. Little evidence exists on the association of physician seniority with patient panel demographics.

## Methods

The Harvard University and University of Minnesota institutional review boards approved this cross-sectional study with a waiver of informed consent because of the use of observational data, in accordance with 45 CFR §46. This study followed the STROBE reporting guideline.

We analyzed 2017 all-payer claims data from athenahealth, Inc, a medical billing and electronic health record vendor, and 2021 Medicare fee-for-service Part B public use files,^3^ the most recent years available. We included physicians with 50 or more patients treated in the period and practices, defined by address, with 4 or more physicians of the same specialty. For each practice, we identified the 2 most junior and 2 most senior physicians using year of medical school graduation, with ties broken randomly. For each physician, we calculated the share of patients treated who were Medicaid (or dually eligible) enrollees and the share from racial and ethnic minority groups (eAppendix 1 in Supplement 1). Physicians were classified into cognitive (eg, primary care or endocrinology), procedural (any surgical- or procedure-predominant specialty), or non–office-based (ie, emergency medicine or radiology) specialties (eTable in Supplement 1). We hypothesized no differences among non–office-based specialists given that these patients and physicians have less choice. We tested for differences in patient panel composition with linear regression models using a dependent outcome of physician-level Medicaid or minority group share of patients and an independent indicator for physician seniority rank (ie, most junior, second most junior, second most senior, and most senior) within each practice, controlling for practice fixed effects (eAppendix 2 in Supplement 1).

## Results

There were 28 895 physicians in 6166 practices with 29 705 902 patients (59.3% White and 40.7% from racial and ethnic minority groups) in athenahealth data and 170 022 physicians in 18 970 practices with 104 588 217 patient-physician pairs (77.1% White and 22.9% from racial and ethnic minority groups) in Medicare data. Median (IQR) experience was 20 (9-34) and 19 (11-29) years, respectively, whereas the median (IQR) practice-level gap (most junior to most senior) was 26 (19-33) and 25 (18-32) years, respectively.

Among cognitive and procedural physicians in athenahealth, the Medicaid share of patients was 17.39% and 15.29% for the most junior vs most senior physician, a seniority gap of −2.10 percentage points (PP) (95% CI, −2.45 to −1.75 PP) (Figure). In Medicare, the seniority gap for Medicaid was 2.25 PP (95% CI, −2.51 to −2.00 PP). The seniority gap in the fraction of minority group patients was −1.36 PP (95% CI, −1.63 to −1.10 PP) in athenahealth and −0.96 PP (95% CI, −1.21 to −0.70 PP) in Medicare.

**Figure.**
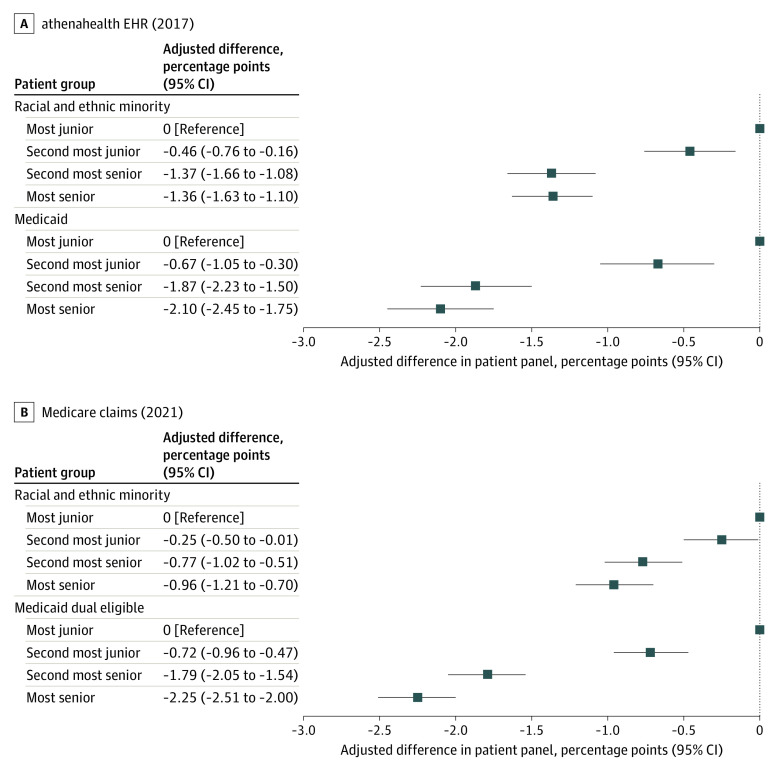
Difference in Medicaid and Minority Group Patient Percentage by Physician Seniority The figure displays coefficients and 95% CIs from 4 separate linear regression analyses for the adjusted difference in the percentage of patients by patient group treated by within-practice seniority. Samples include only office-based physician specialties (ie, cognitive and procedural specialties). Analyses regress Medicaid (or dual eligible) share or racial and ethnic minority group patient share on physician rank (ie, most junior, second most junior, second most senior, and most senior), controlling for practice fixed effects among athenahealth and Medicare samples. The most junior physician is the reference category for physician rank. EHR indicates electronic health record.

By specialty group, the largest Medicare share seniority gaps were in procedural specialties (difference, −2.85 PP [95% CI, −3.34 to −2.35 PP] for athenahealth and −3.24 PP [95% CI, −3.61 to −2.86 PP] for Medicare) (Table). There was no seniority gap by Medicaid or minority group for non–office based specialists.

**Table.** Medicaid and Minority Group Patient Shares by Physician Seniority and Specialty

Patient group, data source, and specialty type	Adjusted marginal patient share estimate, % (95% CI)^a^		Seniority gap, percentage points (95% CI)	*P* value
	Most junior physician	Most senior physician		
Medicaid or dual eligible				
athenahealth				
Cognitive	16.89 (16.62 to 17.15)	15.28 (15.03 to 15.54)	−1.60 (−2.03 to −1.18)	<.001
Procedural	17.15 (16.86 to 17.44)	14.30 (14.00 to 14.59)	−2.85 (−3.34 to −2.35)	<.001
Not office based	22.08 (21.67 to 22.48)	22.45 (21.96 to 22.94)	0.37 (−0.39 to 1.13)	.34
Medicare				
Cognitive	24.80 (24.60 to 25.00)	23.00 (22.79 to 23.20)	−1.80 (−2.13 to −1.47)	<.001
Procedural	18.37 (18.14 to 18.60)	15.13 (14.91 to 15.36)	−3.24 (−3.61 to −2.86)	<.001
Not office based	25.63 (25.39 to 25.88)	25.35 (25.09 to 25.60)	−0.29 (−0.70 to 0.13)	.18
Racial and ethnic minority				
athenahealth				
Cognitive	18.61 (18.42 to 18.81)	17.44 (17.24 to 17.64)	−1.17 (−1.48 to −0.86)	<.001
Procedural	21.04 (20.79 to 21.30)	19.33 (19.10 to 19.56)	−1.71 (−2.11 to −1.31)	<.001
Not office based	12.47 (12.16 to 12.78)	12.44 (12.16 to 12.72)	−0.02 (−0.55 to 0.50)	.93
Medicare				
Cognitive	23.18 (22.98 to 23.38)	22.53 (22.34 to 22.72)	−0.65 (−0.97 to −0.33)	<.001
Procedural	20.37 (20.11 to 20.62)	18.73 (18.49 to 18.98	−1.64 (−2.05 to −1.22)	<.001
Not office based	22.56 (22.30 to 22.83)	22.54 (22.27 to 22.81)	−0.02 (−0.47 to 0.43)	.93

^a^ The table displays marginal effects from regressions identical to those in the Figure but stratified by specialty type.

## Discussion

In 2 independent data sets, this cross-sectional study found that senior physicians treated fewer traditionally underserved patients than their junior colleagues within practices. This gap was not present for specialties without scheduled patient visits, suggesting that physician- or practice-level incentives, such as lower reimbursement rates or greater administrative hassles for Medicaid enrollees, may contribute.^4^ Some differences may also be associated with differences in patient choice or agency. An important limitation is that this analysis is observational, so results should be interpreted as hypothesis-generating associations.

The potential clinical impact of this segregation and its importance for disparities in care require further investigation. Existing literature suggests a mixed association between physician tenure in medicine and patient outcomes.^5,6^ However, to the extent that patients value seeing more experienced physicians, disparities in access are a concerning barrier to equitable care.

## Notes

Supplement 1. **eAppendix 1.** Patient Race and Ethnicity Variables **eTable.** Specialty Categories **eAppendix 2.** Statistical Analysis Details

Supplement 2.
Data Sharing Statement
