# Supplementary material for: Differences by Physician Seniority in Race and Ethnicity and Insurance Coverage of Treated Patients
Source: JAMA Netw Open. 2023 Dec 13;6(12):e2347367. doi: 10.1001/jamanetworkopen.2023.47367 (PMC10719748; doi:10.1001/jamanetworkopen.2023.47367)
Supplement: Supplement 1. — eAppendix 1. Patient Race and Ethnicity Variables eTable. Specialty Categories eAppendix 2. Statistical Analysis Details [file jamanetwopen-e2347367-s001.pdf]

# Supplemental Online Content

Neprash HT, Chan DC, Mehrotra A, Barnett ML. Differences by physician seniority in race and ethnicity and insurance coverage of treated patients. *JAMA Netw Open*. 2023;6(12):e2347367. doi:10.1001/jamanetworkopen.2023.47367

**eAppendix 1.** Patient Race and Ethnicity Variables

**eTable.** Specialty Categories

**eAppendix 2.** Statistical Analysis Details

This supplemental material has been provided by the authors to give readers additional information about their work.

© 2023 Neprash HT et al. *JAMA Network Open.*

**eAppendix 1.** Patient Race and Ethnicity Variables

For patients present in the athenahealth and Medicare claims data, we classified them as members of racial and ethnic minority groups using available information on patient race and ethnicity. In the athenahealth data, race and ethnicity variables rely on patient-reported information collected when a patient is first treated by a clinician-client of athenahealth. We used this information to construct a binary patient-level minority group indicator equal to zero for patients with missing data and patients self-identifying as non-Hispanic White; one otherwise.

In Medicare data, we relied on information about patient race and ethnicity deriving from enrollment data. Again, this information is self-reported by Medicare beneficiaries, at the time of Medicare enrollment. To improve information for the Asian/Pacific Islander and Hispanic groups (who may not have been captured in the original response categories, depending on year of Medicare enrollment), the Centers for Medicare and Medicaid services applies an algorithm developed by the Research Triangle Institute.

Because of small sample sizes of racial and ethnic minority groups within physician panels in the Medicare data, many physicians had suppressed data for racial/ethnic minority groups. To overcome this missing data problem, we aggregated the groups into two categories of non-Hispanic white ("white") and racial/ethnic minority (Black, Hispanic, Asian/Pacific Islander, Native American, and other; as classified by Medicare). In the athenahealth data, the same groups were made, white and racial/ethnic minority (Black, Asian, Native Hawaiian and Pacific Islander, Other, Missing/Patient Declined as captured in athenahealth data).

© 2023 Neprash HT et al. *JAMA Network Open.*

**eTable.** Specialty Categories

| Specialty Category | Specialty Code | Description |
|---|---|---|
| Cognitive | 1 | General practice |
| | 3 | Allergy/immunology |
| | 6 | Cardiology |
| | 7 | Dermatology |
| | 8 | Family practice |
| | 11 | Internal medicine |
| | 12 | Osteopathic manipulative therapy |
| | 13 | Neurology |
| | 17 | Hospice and Palliative Care |
| | 21 | Cardiac Electrophysiology |
| | 25 | Physical medicine and rehabilitation |
| | 26 | Psychiatry |
| | 27 | Geriatric Psychiatry |
| | 29 | Pulmonary disease |
| | 37 | Pediatric medicine |
| | 38 | Geriatric medicine |
| | 39 | Nephrology |
| | 44 | Infectious disease |
| | 46 | Endocrinology |
| | 66 | Rheumatology |
| | 79 | Addiction medicine |
| | 81 | Critical care (intensivists) |
| | 82 | Hematology |
| | 83 | Hematology/oncology |
| | 84 | Preventive medicine |
| | 86 | Neuropsychiatry |
| | 90 | Medical oncology |
| | C0 | Sleep medicine |
| | C3 | Interventional cardiology |
| | C7 | Advanced heart failure and transplant cardiology |
| | C9 | Hematopoietic cell transplantation |
| Non-Office | 5 | Anesthesiology |
| | 22 | Pathology |
| | 30 | Diagnostic radiology |
| | 93 | Emergency medicine |
| | 94 | Interventional radiology |
| | C6 | Hospitalist |
| | C8 | Medical toxicology |
| Procedural | 2 | General surgery |
| | 4 | Otolaryngology |
| | 9 | Interventional Pain Management |
| | 10 | Gastroenterology |
| | 14 | Neurosurgery |
| | 16 | Obstetrics/gynecology |
| | 18 | Ophthalmology |
| | 20 | Orthopedic surgery |
| | 23 | Sports medicine |
| | 23 | Peripheral vascular disease, medical |
| | 24 | Plastic and reconstructive surgery |
| | 28 | Colorectal surgery |
| | 33 | Thoracic surgery |
| | 34 | Urology |
| | 40 | Hand surgery |
| | 72 | Pain Management |
| | 76 | Peripheral vascular disease |
| | 77 | Vascular surgery |
| | 78 | Cardiac surgery |
| | 91 | Surgical oncology |
| | 92 | Radiation oncology |
| | 98 | Gynecologist/oncologist |

© 2023 Neprash HT et al. *JAMA Network Open.*

**eAppendix 2.** Statistical Analysis Details

Analyses were conducted using Stata version 17 (StataCorp) and R v4.1.2, with statistical significance defined as 2-sided $P < .05$.

© 2023 Neprash HT et al. *JAMA Network Open.*
