# Supplementary material for: Differences by Physician Seniority in Race and Ethnicity and Insurance Coverage of Treated Patients
Source: JAMA Netw Open. 2023 Dec 13;6(12):e2347367. doi: 10.1001/jamanetworkopen.2023.47367 (PMC10719748; doi:10.1001/jamanetworkopen.2023.47367)
Supplement: Supplement 2. — Data Sharing Statement [file jamanetwopen-e2347367-s002.pdf]

Data Sharing Statement

Neprash. Differences by Physician Seniority in Race and Ethnicity and Insurance Coverage of Treated Patients. *JAMA Netw Open*. Published online December 13, 2023. doi:10.1001/jamanetworkopen.2023.47367

Data
Data available: No
